# Long-Term Test-Retest Reliability of Auditory Gamma Oscillations Between Different Clinical EEG Systems

**DOI:** 10.3389/fpsyt.2020.00876

**Published:** 2020-09-02

**Authors:** Yoji Hirano, Itta Nakamura, Shunsuke Tamura, Toshiaki Onitsuka

**Affiliations:** ^1^ Department of Neuropsychiatry, Graduate School of Medical Sciences, Kyushu University, Fukuoka, Japan; ^2^ Neural Dynamics Laboratory, Research Service, VA Boston Healthcare System, Boston, MA, United States; ^3^ Department of Psychiatry, Harvard Medical School, Boston, MA, United States

**Keywords:** auditory steady-state response (ASSR), electroencephalogram (EEG), gamma oscillation, test-retest reliability, schizophrenia

## Abstract

**Objective:**

There is increasing interest in the utility of gamma-band activity for assessing various brain functions, including perception, language, memory, and cognition. The auditory steady-state response (ASSR) involves neural activity in the brain elicited by trains of a click sound, and its maximum response is obtained at 40 Hz (40-Hz ASSR). Abnormalities of the 40-Hz ASSR are also widely reported in patients with schizophrenia. Thus, the test-retest reliability of the ASSR is important for its clinical and translational application. However, there are only limited studies reporting the short-term reliability between acquisitions at two time points made using the same electroencephalogram (EEG) system. Furthermore, the long-term reliability between multiple EEG systems and the reliability of spontaneous gamma activity are unknown but are crucial for multicenter collaborative research.

**Methods:**

We examined the long-term test–retest reliability of 40-Hz ASSR oscillatory activities indexed by the phase locking factor (PLF), evoked power, and (non-phase-locked) induced power between two clinical 19-electrode EEG systems [recorded twice for EEG-1 (time1 and time2) and EEG-2 (time3 and time4)] at four time points from 14 healthy controls over a duration of 5 months. Test-retest reliability was examined using intraclass correlation coefficients (ICCs).

**Results:**

Both PLF and evoked power showed good to excellent ICCs (>0.60), mainly in the Fz-electrode, both within each EEG system—EEG-1 [(time1 vs. time2) PLF: ICC = 0.66, evoked power: ICC = 0.88] and EEG-2 [(time3 vs. time4) PLF: ICC = 0.82, evoked power: ICC = 0.77]—and between the two EEG systems [(EEG-1 vs. EEG-2) PLF: ICC = 0.73, evoked power: ICC = 0.84]. In contrast, induced power showed the highest (excellent) ICC between the two EEG systems (ICC = 0.95) mainly in the Cz-electrode. For PLF, the Fz-electrode showed better test-retest reliability across all EEG recordings than the Cz-electrode (Fz: ICC = 0.67, Cz: ICC = 0.63), whereas we found similar excellent reproducibility across all EEG recordings from both electrodes for evoked power (Fz: ICC = 0.79, Cz: ICC = 0.77) and induced power (Fz: ICC = 0.79, Cz: ICC = 0.80).

**Conclusion:**

The 40-Hz ASSR oscillatory activities, including induced power, showed excellent test-retest reliability, even when using different EEG systems over a duration of 5 months. These findings confirm the utility of the 40-Hz ASSR as a reliable clinical and translatable biomarker for multicenter collaborative research.

## Introduction

Gamma-band (30−100 Hz) neural oscillations are implicated in normal perception, cognition, and memory, while recent empirical studies suggest that gamma-band activity is disrupted in patients with schizophrenia (1). The gamma-aminobutyric acid (GABA) transmission system plays an important role in gamma-band oscillations, and GABAergic interneurons, especially GABAergic parvalbumin-expressing interneurons, can control the firing timing of pyramidal neurons (2). GABAergic inhibitory interneurons also play a critical role as rhythmic pacemakers by generating regular inhibitory postsynaptic potentials to excitatory glutamatergic pyramidal neurons (3). The reciprocal balance between excitability and inhibition is essential for the generation of gamma-band activity (4). Normal neuronal information processes rely on a functional excitability/inhibitory balance (E/I-balance), while failure to maintain this balance is hypothesized to cause abnormalities in gamma-band activity in patients with schizophrenia. Thus, such abnormalities may be the neurophysiological basis of sensory processing deficits in this disorder (5, 6). Among the neural oscillation indices, the auditory steady-state response (ASSR), which can elicit strong synchronous activities with the corresponding stimulus frequency during constant click sounds, is useful in evaluating gamma-band activity (7).

Many studies have shown a decreased evoked power value and phase synchronization of 40-Hz ASSR in both the early and chronic phases of schizophrenia (8–13). In addition to decreased stimulus-locked ASSR activities, recent reports show increased non-phase-locked spontaneous gamma-band activity (induced power) during click-sound stimulation in schizophrenia patients (9), along with cortical volume deficits in the primary auditory cortex (14). These data suggest that both the reduction of ASSR and the abnormal noisy background gamma-band activity may contribute to the pathology of this disease. Moreover, our previous findings (9) resembled the increased spontaneous broadband gamma power that is often reported in animal models of schizophrenia based on N-methyl-d-aspartate receptor (NMDAR) hypofunction and E/I-imbalance [e.g., (15)]. Hence, from the translational research perspective, it is critical to validate the robustness of the spontaneous gamma power.

Other mental conditions such as bipolar disorder or affective psychosis show similar ASSR deficits, indicating that ASSR may provide a useful index for distinguishing these conditions from major depressive disorder (12, 16). However, the utility of ASSR as a biomarker remains limited by the small sample size (<100) in these studies. Thus, in addition to conducting detailed neurophysiological studies at a single site, further multisite studies on the cross-disease ASSR features of large samples are required to develop the framework for future clinical applications.

To achieve such large-sample collaborative studies, it is critical to confirm the repeatability and reproducibility of the ASSR. Legget et al. confirmed the reproducibility of the 40-Hz ASSR within the same device using an electroencephalogram (EEG) and a magnetoencephalogram (MEG) in healthy controls (17), while Roach et al. reported the reproducibility of 40-Hz ASSR in patients with schizophrenia (18). However, the reproducibility between different EEG devices is unknown. Various factors that influence EEG recordings, such as amplifier and filter systems, electrode type, and sampling frequency, differ between EEG systems. Furthermore, collaborative neuroimaging MRI studies reported marked variations in brain volume measurements related to the characteristics of different scanners (19, 20). Similarly, there are different measurement programs (e.g., montage, sampling rate, band-pass filters, and impedance) and amplifier structures for EEG recording. Thus, it is also critical to confirm reproducibility between different EEG systems. More importantly, previous test-retest reliability studies of 40-Hz ASSR are limited by the durations used, with just two time points spaced about one week apart. Longitudinal evaluation of neurophysiological indices is receiving more attention for the detection of biological markers around the onset of schizophrenia, including in subjects with a high clinical risk (21–23). Therefore, long-term test-retest reliability studies of multi-time point recordings are crucial for providing evidence of the reproducibility of 40-Hz ASSR as a stable clinical biomarker.

Therefore, in the present study, we examined the long-term test-retest reproducibility of 40-Hz ASSR oscillatory activities, including induced power, by performing two EEG oscillatory measurements at four time points using two EEG systems to assess the utility of the 40-Hz ASSR index.

## Methods and Materials

### Participants

Fourteen healthy adults (mean age: 37.7 ± 8.6 years; six men, eight women) participated in this study. All participants were screened for normal hearing. The inclusion criteria were: 1) no history of neurological illness including epilepsy, 2) no history of major head trauma, 3) no history of alcohol/drug dependence or abuse, 4) no history of electroconvulsive therapy, and 5) verbal IQ > 75. Participants were also screened using the Structured Clinical Interview (non-patient edition), and no subjects or their first-degree relatives were found to have an Axis-I psychiatric disorder. All subjects were recruited from the local community in Fukuoka metropolitan areas. Experimental procedures were approved by the Kyushu University Institutional Review Board for Clinical Trials (approval number 27-207) and conformed to the Declaration of Helsinki. Before the experiments, written informed consent was acquired from each subject after a detailed explanation of the study.

### EEG Recording and Procedures

To examine the long-term test-retest reproducibility of the 40-Hz ASSR using different EEG systems, EEG measurements at four time points were repeatedly performed with two EEG systems (EEG1: EEG-1000; Nihon-Koden Co., Tokyo, Japan; EEG2: EEG-1200; Nihon-Koden Co., Tokyo, Japan). The interval times between the four EEG measurements were: time1−time2 = 31.2 ± 26.9 days, time2−time3 = 73.0 ± 34.9 days, and time3−time4 = 42.7 ± 28.9 days. Hence, the EEG data were collected four times over a duration of around 5 months. The two EEG systems used the same electrode format and number but had different methods for measuring the time constant (TC; EEG-1, 10 s; EEG-2, 2 s) and different anti-aliasing filter systems. All continuous EEG data were acquired using a 19-electrode silver/silver chloride (Ag–AgCl) passive electrode (Nihon-Koden Co.) with a standard 10–20 system electrode placement (24). Electrodes were placed on the outer canthi and the supra-orbit of the right eye to assess eye movements. Impedances were <20 kV at all sites. EEG recordings were amplified using Neurofax amplifiers (Nihon-Koden Co.) and digitized at 500 Hz with a bandpass filter of 1–200 Hz. Subjects were instructed to close their eyes and relax in the supine position in a shielded room free from external interference or noise. Spontaneous EEGs were recorded for 4 min, followed by 3 min of ASSR recording. The ASSR stimuli consisted of 150 × 40 Hz trains of 1-ms white noise clicks (500-ms duration, 600-ms inter-train interval), which were presented binaurally through inner earphones (80-dB sound pressure level).

### Data Preprocessing

EEG data were preprocessed using MNE Python (https://mne.tools/dev/index.html). An offline 1–100 Hz bandpass filter and notch filters of 60 Hz were applied to the EEG data. Ocular and cardiac artifacts were removed using independent component analysis. Artifact-free EEG data were average-referenced, and epochs of 1,100 ms were created, starting at 400 ms prior to stimulus onset and lasting for 700-ms post-stimulus onset (epochs with peak-to-peak amplitude > 200 mV were rejected). The number of accepted trials used for further analyses were: time1, 149.7 ± 0.5; time2, 149.5 ± 0.5; time3, 145.4 ± 1.2; time4, 146.1 ± 1.0. The 40-Hz ASSR was analyzed using the Morlet waveform transform (f_0_/σ_f_ = 7). We calculated the phase locking factor (PLF), evoked power, and induced (non-phase-locked) power that were applied to the single-trial epochs in 0.1-Hz steps from 4−100 Hz at each time point from −400 ms (prior to stimulus onset) to 700 ms (post-stimulus onset). PLF measures the variance of phase across single trials and ranges from 0 (random distribution) to 1 (perfect phase locked). Evoked power measures the power of the average evoked potential in which the contribution of non-stimulus locked activity is minimized. Induced power was measured by subtracting the evoked power from the averaged power of the single-trial potential. The mean PLF and evoked power from 100 to 500 ms were averaged across 10 Hz bands (35–45 Hz). The mean (non-phase-locked) induced power in the baseline period (−400–0 ms) was calculated by averaging the power across 70-Hz bands (30–100 Hz).

### Statistical Analyses

In EEG studies, ASSR has a maximum response near Fz, and the Fz or FCz electrode is widely used (13, 25). Thus, we used the Fz electrodes for the main statistical analyses, while secondary analyses were performed to determine which electrodes (Fz or Cz) had better reproducibility. First, to assess the reproducibility within the same EEG system, we calculated intraclass correlation coefficients (ICCs) (26) between time1 and time2 (recorded by EEG-1) and between time3 and time4 (recorded by EEG-2) for the mean PLF, evoked power, and induced power in the Fz electrode and Cz electrode. Second, to assess the reproducibility between the two EEG systems (EEG-1 vs. EEG-2), we calculated the ICCs between the response in EEG-1 (averaged PLF and evoked power across time1 and time2) and response in EEG-2 (averaged PLF and evoked power across time3 and time4) in the Fz and Cz electrodes. Furthermore, the reliability of the PLF, evoked power and induced power of the Fz and Cz electrodes across four time points for both EEG systems were also examined using ICCs. We judged the quality of the ICCs as follows: ICC < 0.4 poor, 0.40–0.60 fair, 0.60–0.75 good, and 0.75–1 excellent (27).

## Results

**Figure 1:**
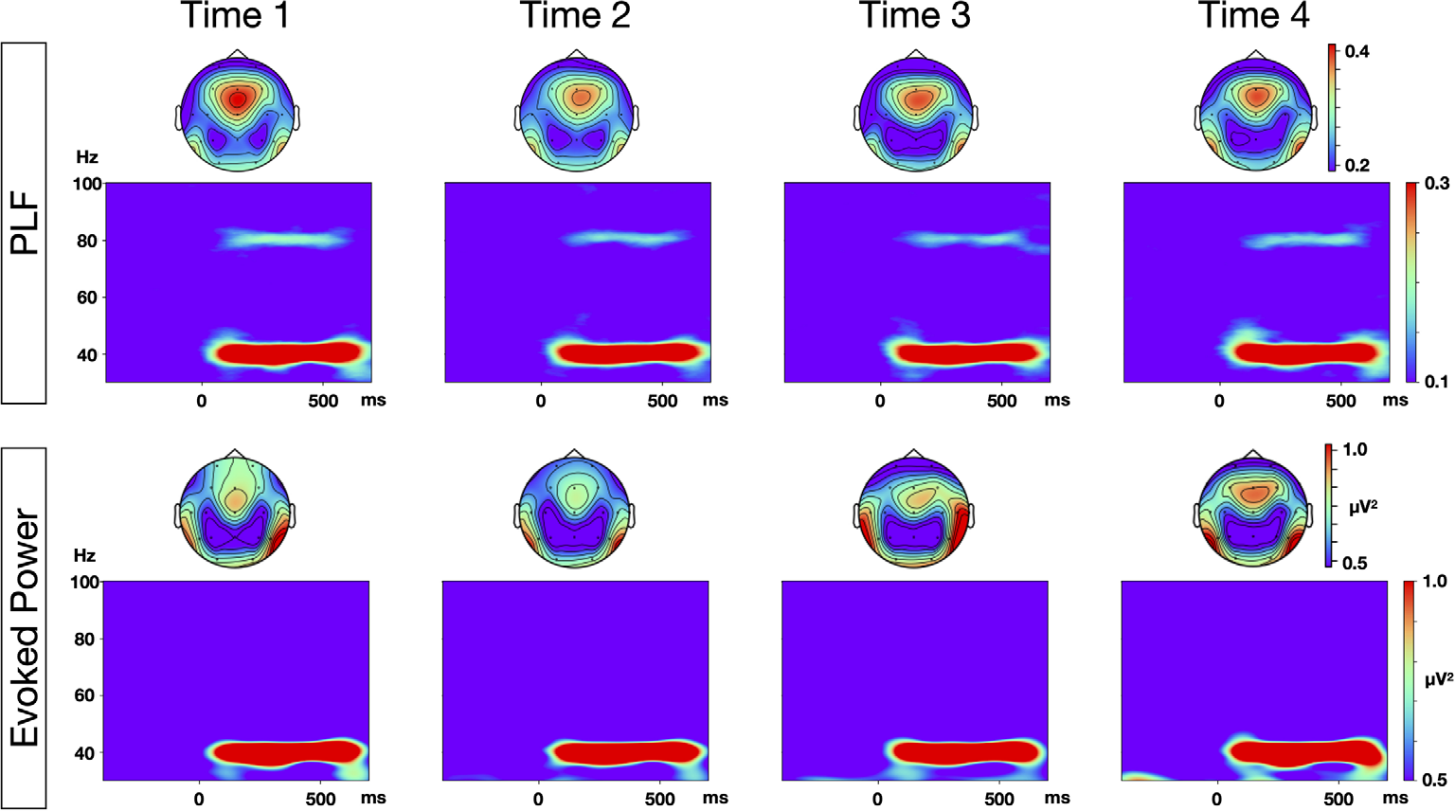
Time-frequency maps of phase locking factor (PLF) and evoked power at each time points. Grand average time-frequency maps at Fz electrode, and topographies of PLF and evoked power within the range of 35–45 Hz, at each of the four time points.

**Figure 2:**
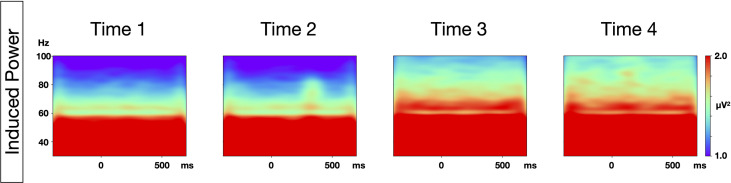
Time-frequency maps of induced power at each time point. Grand average time-frequency maps at the Cz electrode at each of the four time points.

The grand average time-frequency maps at the Fz electrode, and the topography of PLF and evoked power at each time point are shown in **Figure 1**. The distribution of the 40-Hz ASSR activity topography was largely similar at the four different time points. The topography maps confirmed that the highest 40-Hz ASSR activities at each time point, and the highest oscillatory measures (PLF and evoked power), were located around the Fz-electrode. The grand average time-frequency maps of the induced power at the Cz electrode at each time point are shown in **Figure 2**. The induced powers in the gamma range measured by EEG-1 seemed to be smaller than those measured by EEG-2.

Both PLF and evoked power (phase-locked activities) showed good to excellent ICCs (> 0.60), mainly in the Fz-electrode, within both EEG-1 [(time1 vs. time2) PLF: ICC = 0.66, evoked power: ICC = 0.88] and EEG-2 [(time3 vs. time4) PLF: ICC = 0.82, evoked power: ICC = 0.77]. Additionally, the Fz-electrode showed higher ICCs than the Cz-electrode for PLF (Fz: ICC = 0.73, Cz: ICC = 0.63) and evoked power (Fz: ICC = 0.84, Cz: ICC = 0.78) between the two EEG systems (EEG-1 vs. EEG-2).

Induced power (non-phase-locked activity) showed excellent ICCs (> 0.75) in both electrodes within both EEG-1 [(time1 vs. time2) Fz: ICC = 0.75, Cz: ICC = 0.91] and EEG-2 [(time3 vs. time4) Fz: ICC = 0.92, Cz: ICC = 0.74]. Moreover, in contrast to PLF and evoked power, the Cz-electrode showed the highest ICC for induced power between the two EEG systems [(EEG-1 vs. EEG-2) Fz: ICC = 0.73, Cz: ICC = 0.95].

Similarly, in the test to evaluate reliability across all EEG recordings, the Fz-electrode showed higher test-retest reliability than the Cz-electrode for PLF (Fz: ICC = 0.67, Cz: ICC = 0.63). In contrast, we found similar excellent test-retest reliability across all EEG recordings in both electrodes for evoked power (Fz: ICC = 0.79, Cz: ICC = 0.77) and induced power (Fz: ICC = 0.79, Cz: ICC = 0.80).

**Figure 3:**
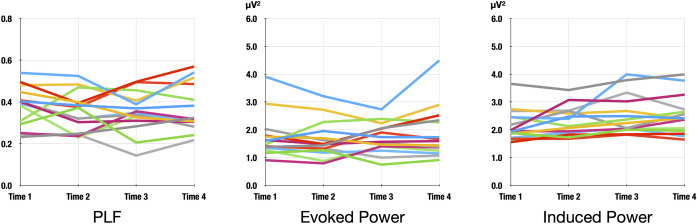
Line plots of PLF, evoked power, and induced power at the four time points.

A summary of the statistical results is shown in **Table 1**, and line plots of PLF, evoked power, and induced power at the four time points are shown in **Figure 3**, which shows that compared with PLF and evoked power, induced power showed only small fluctuations throughout the four recording time points.

**Table 1:** Summary of the test-retest reliability indexed by intraclass correlation coefficient.

Oscillatory measures	Electrode	ICC between each EEG recordings at different time points		ICC between two different EEG systems	ICC across all time points and EEG systems
		Time 1 vs. Time 2(EEG-1)	Time 3 vs. Time 4(EEG-2)	EEG-1 vs. EEG-2(averaged across each time points)	
PLF	Fz	0.66	**0.82**	0.73	0.67
	Cz	0.64	**0.87**	0.63	0.63
Evoked Power	Fz	**0.88**	**0.77**	**0.84**	**0.79**
	Cz	**0.80**	**0.92**	**0.78**	**0.77**
Induced Power	Fz	**0.75**	**0.92**	0.73	**0.79**
	Cz	**0.91**	0.74	**0.95**	**0.80**

ICC, intraclass correlation coefficient. ICC is greater than 0.75 are in bold font.

## Discussion

To the best of our knowledge, this is the first report of long-term excellent test-retest reliability at multiple time points for 40-Hz ASSR oscillatory measurements. These findings are in line with previous reports (17, 18, 28) demonstrating a good test-retest reliability of 40-Hz ASSR measures at two time points. However, we also provide new evidence that the 40-Hz ASSR measurement, including baseline induced power, has excellent long-term test-retest reliability (stable over four time points covering a 5-month period) between different EEG systems, which is crucial for its use as a stable clinical biomarker in future collaborative research. Given that longitudinal evaluations of neurophysiological EEG/MEG indices have attracted much attention as biological markers for detecting the onset of schizophrenia and subjects at a high clinical risk (21–23), our results showing the long-term excellent test-retest reliability of 40-Hz ASSR oscillatory measurements provide promising evidence that they can be used as a robust clinical biomarker for the early detection of schizophrenia.

The reproducibility of the 40-Hz ASSR in the present study was similar or better than that reported for other event-related potential components used in psychiatric research [e.g., the P300 amplitude (*r* or *ρ* = 0.31−0.81), the N1 amplitude (*r* or *ρ* = 0.09−0.75) (29–32), and the mismatch negativity (confidence levels = 0.3−0.6)] (33–37). It should be noted that most of these event-related potential studies were performed using high-quality research EEG systems (non-medical applications), whereas our study showed excellent reproducibility of the 40-Hz ASSR with a normal clinical EEG system. When conducting multisite EEG studies with large-scale samples, it is critical to use such clinical EEG systems present in the general clinical setting.

Importantly, our results also demonstrated that the Fz electrode had the highest reliability or internal consistency for both PLF and evoked power when evaluating the 40-Hz ASSR using the 19-electrode standard 10–20 clinical EEG system. Our results are comparable with previous reports of excellent test-retest reliability of the 40-Hz ASSR at the Fz or FCz electrodes (18, 28). In contrast, the Cz-electrode showed the highest ICC for the induced power between the two EEG systems. A potential explanation for these differences may arise from the different spatial distributions over the electrodes, although this needs to be confirmed in future studies. Thus, given these findings and because there is no FCz electrode in the 10–20 clinical EEG system, for 40-Hz ASSR analysis, Fz may be the optimal electrode to analyze the PLF and evoked power, whereas Cz may be more stable for analyzing induced power in a 19-electrode clinical EEG system.

An alternative analysis for evaluating ASSR is to apply the source estimation. However, in our test-retest reliability study, we did not perform source level analysis because our clinical EEG systems consisted of only 19 electrodes, which was insufficient for source level analysis. Nevertheless, certain task-related EEG measures appear to benefit from source level analysis, with improved signal-to-noise ratio and test-retest reliability (28, 38, 39). In terms of sensor-level analysis, our results are comparable with a study by McFadden et al. who reported a fine reproducibility of ASSR at the FCz electrode (PLF, *r* = 0.89; normalized evoked power, *r* = 0.90). In that study, the authors also conducted source level signal-space projection analysis within the auditory cortex and found a similar test-retest reliability (left PLF, *r* = 0.90; left normalized evoked power, *r* = 0.70; right PLF, *r* = 0.69; right normalized evoked power, *r* = 0.40). Recently, Roach et al. demonstrated fine reproducibility of the 40-Hz ASSR at the Fz electrode using the G-coefficient in both healthy subjects and schizophrenia patients (18). Interestingly, in that study, there was fine test retest reliability of the ASSR in schizophrenia patients, which to some extent was better than that of healthy controls. Given these findings, we suggest that sensor level analysis is sufficient for ASSR-EEG settings as long as the evaluation of activity in specific regions (such as both auditory cortices) is not required.

In contrast with EEG, MEG has a greater spatial resolution and is more suited to detect detailed local- and whole-brain activity or long-range connections in cortical networks. However, MEG is currently unsuitable for daily clinical use as a versatile EEG system because of its complicated technical demands and high expense, including the requirement for specialized shielding and helium. Legget et al. compared test-retest reliability of the 40-Hz ASSR between EEG and MEG, and demonstrated similar reliability for both systems, suggesting that either measure can be reliably employed in ASSR studies (17). Given these findings, we suggest that EEG-measured ASSR is best suited for daily clinical use and for multicenter collaborations.

With regard to the differences in test-retest reliability of each of the ASSR oscillatory measurements, evoked power had better reliability than PLF in our study, which was inconsistent with previous studies (28, 40). Potential explanations for these differences may relate to differences in the systems, which can influence the signal-to-noise ratio in recorded data [e.g., electrode type, amplifier type, and device (EEG vs. MEG)]. Additionally, variability in the distributions of both values between different studies may contribute to these reliability differences. Moreover, to our surprise, induced power showed the highest excellent test-retest reliability between the two EEG systems (ICC = 0.95). Importantly, compared with PLF and evoked power, the induced power showed only small fluctuations throughout the recorded time (**Figure 3**). Given the importance of using gamma power abnormalities as evidence of NMDA receptor hypofunction and E/I-imbalance in schizophrenia, and given our previous work demonstrating increased induced gamma power during ASSR in schizophrenia (9), our findings of the long-term stability of this induced power may add valuable evidence for the use of the 40-Hz ASSR as a robust clinical biomarker of schizophrenia.

There are pros and cons regarding different stimuli used in the ASSR paradigm. Historically, amplitude modulated (AM) tone is widely used in audiology and was found to be more pleasant and less affected by subjects’ attention than the click trains (41). By contrast, Legget et al. reported that all oscillatory indices in response to 40-Hz click train stimuli were more reliable than to 40-Hz white noise AM stimuli across sessions (17). Similarly, McFadden et al. reported that 40-Hz click train stimuli elicit a more reliable estimation of the 40-Hz ASSR than for white noise AM stimuli, probably because of the higher signal-to-noise ratio observed in the click paradigm [e.g., inter-trial coherence (ITC)-click, 2.25 vs. ITC-AM, 1.82]. Griskova et al. also found larger effect sizes for the 40-Hz click train than for the 40-Hz AM stimuli and concluded that the click train provided better differentiation of schizophrenia patients compared with healthy controls (42). Thus, from the viewpoint of reliability and overall clinical utility, the click train stimuli, which were employed in our study, seems the best suited for the clinical settings.

Enhancing attention to the ASSR task reportedly increased the 40-Hz ASSR oscillatory indices (43, 44), while an increased arousal level was demonstrated to reduce the 40-Hz ASSR oscillatory indices (42). More recently, by controlling subjects’ attention, the 40-Hz ASSR in a passive healing condition exhibited excellent reliability in both healthy controls and schizophrenia patients, while an auditory oddball condition showed slightly reduced ASSR reliability (18). Therefore, the simple passive hearing condition used in our ASSR study is suitable in the clinical setting, where some patients may have difficulties in maintaining attention.

A potential caveat in our study is that both EEG systems were from the same company, and although there are differences such as the time constant and filter system, the basic amplifier structure and electrode type were the same. Thus, there is a need to test reproducibility between entirely different EEG systems, to eliminate some of the variability that could stem from using different companies’ EEG systems. Another potential limitation is that our study design could not randomly assign EEG-1 and EEG-2 during the four time points because of the replacement. Nevertheless, our results indicate that switching from one EEG system to its next generation one within the same environment (same stimulus presentation setup and other aspects of the setting) has little to no effect on the ASSR-EEG data recorded. Recently, large-sample collaborative studies are becoming crucial to characterize healthy and clinical population groups in psychiatric research, and reproducibility tests at different centers using traveling subjects would confirm our results. Importantly, if traveling subjects are not available, multicenter studies may also greatly benefit from adapting statistical models correcting for the effects of each site (e.g., 19). It should be noted that by taking advantage of the fact that only one EEG company (Nihon Kohden) is approved for clinical use in Japan, we can conduct large-sample collaborative EEG studies using the same or very similar EEG systems (with the same stimulus presentation, recording environment, and data collection procedures) in Japan. To utilize 40-Hz ASSR as a stable clinical biomarker, we suggest that future studies using different EEG systems from different companies, and harmonized multicenter EEG studies, are required.

## Conclusions

Our findings confirm the excellent long-term reliability and utility of 40-Hz ASSR oscillatory activities, including induced power, even when different EEG systems were used. Thus, we propose that 40-Hz ASSR oscillatory measures are one of the best neurophysiological indices for future collaborative research, which is essential for clinical and translational applications.

## Data Availability Statement

The raw data supporting the conclusions of this article will be made available by the authors, without undue reservation.

## Ethics Statement

The studies involving human participants were reviewed and approved by Kyushu University Institutional Review Board for Clinical Trials. The patients/participants provided their written informed consent to participate in this study.

## Author Contributions

YH and IN had full access to all of the data in the study, and take responsibility for the integrity of the data and the accuracy of the data analysis. All authors were involved in concept and design and performed critical revision of the manuscript for important intellectual content. IN, ST, and YH performed acquisition, analysis, or interpretation of the data, drafted the manuscript, and performed statistical analysis. TO and YH performed supervision.

## Funding

This work was supported by JSPS KAKENHI [Grant No. JP18K15519 (IN), JP15K09836 (YH), JP18K07604 (YH), JP19H03579 (YH), and JP19H00630 (YH)] and in part by AMED under Grant Number JP20dm0207069 (TO). This work was also partly supported by the Fund for Pharmacopsychiatry Research from the Senshin Medical Research Foundation (YH), Fund for Takeda Science Foundation (YH), Research Grant Award (YH) from UBE Industries Foundation, and the SIRS Research Fund Award (YH).

## Conflict of Interest

The authors declare that the research was conducted in the absence of any commercial or financial relationships that could be construed as a potential conflict of interest.
